# Towards translational therapies for multiple system atrophy

**DOI:** 10.1016/j.pneurobio.2014.02.007

**Published:** 2014-07

**Authors:** Daniela Kuzdas-Wood, Nadia Stefanova, Kurt A. Jellinger, Klaus Seppi, Michael G. Schlossmacher, Werner Poewe, Gregor K. Wenning

**Affiliations:** a Department of Neurology, Innsbruck Medical University, Anichstraße 35, Innsbruck 6020, Austria; b Institute of Clinical Neurobiology, Vienna, Austria; c Divisions of Neuroscience and Neurology, The Ottawa Hospital Research Institute, University of Ottawa, 451 Smyth Road, RGH #1412, Ottawa, ON, K1H 8M5, Canada

**Keywords:** MSA, multiple system atrophy, MSA-P, parkinsonian variant of MSA, MSA-C, cerebellar variant of MSA, α-Syn, alpha-synuclein, SND, striatonigral degeneration, OPCA, olivopontocerebellar atrophy, GCI, (oligodendro-)glial cytoplasmic inclusions, UMSARS, unified MSA rating scale, wt, wild type, tg, transgenic, PLP, proteolipid protein, MBP, myelin basic protein, CNP, 2′,3′-cyclic-nucleotide 3′-phosphodiesterase, SN, substantia nigra, Multiple system atrophy, Striatonigral degeneration, Olivopontocerebellar atrophy, Alpha-synuclein, Neurodegeneration

## Abstract

Multiple system atrophy (MSA) is a fatal adult-onset neurodegenerative disorder of uncertain etiopathogenesis manifesting with autonomic failure, parkinsonism, and ataxia in any combination. The underlying neuropathology affects central autonomic, striatonigral and olivopontocerebellar pathways and it is associated with distinctive glial cytoplasmic inclusions (GCIs, Papp-Lantos bodies) that contain aggregates of α-synuclein. Current treatment options are very limited and mainly focused on symptomatic relief, whereas disease modifying options are lacking. Despite extensive testing, no neuroprotective drug treatment has been identified up to now; however, a neurorestorative approach utilizing autologous mesenchymal stem cells has shown remarkable beneficial effects in the cerebellar variant of MSA. Here, we review the progress made over the last decade in defining pathogenic targets in MSA and summarize insights gained from candidate disease-modifying interventions that have utilized a variety of well-established preclinical MSA models. We also discuss the current limitations that our field faces and suggest solutions for possible approaches in cause-directed therapies of MSA.

## Highlights

• This review summarizes the progress made in MSA research during the past decade.
• Pre-motor features have gained relevance as early diagnostic markers.
• Animal models have been exploited to discover novel interventional targets.
• Candidate α-synuclein lowering therapies have been explored in preclinical models.

## 1 Introduction

**Fig. 1:**
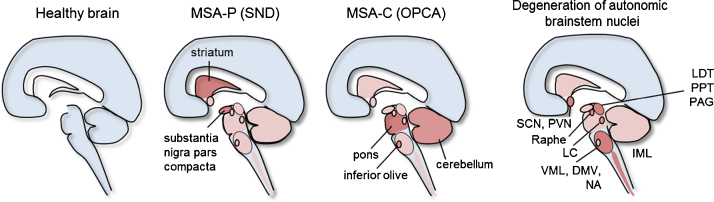
Neuropathology underlying MSA-P, MSA-C and autonomic failure in MSA. Striatonigral degeneration is the underlying pathology of MSA-P, olivopontocerebellar atrophy occurs in MSA-C and degeneration of autonomic brainstem nuclei plays a role for characteristic autonomic failure in MSA patients. SND, striatonigral degeneration; OPCA, olivopontocerebellar atrophy; SCN, suprachiasmatic nucleus; PVN, paraventricular nucleus; LC, locus coeruleus; VML, ventrolateral medulla; DMV, dorsal motor nucleus of the vagus; NA, nucleus ambiguus; IML, intermediolateral column of the thoracic spinal cord; LDT, laterodorsal tegmental nucleus; PPT, pedunculopontine tegmental nucleus; PAG, periaqueductal gray.

Multiple system atrophy is an adult onset, fatal, neurodegenerative disease, presenting with autonomic failure, parkinsonism, and cerebellar ataxia in different combinations. The mean age of disease onset is ∼57 years and survival after disease onset lies between six and nine years (Stefanova et al., 2009a). The prevalence is 1.9–4.9/100,000 and the incidence is 3/100,000/year in the population over 50 years, MSA therefore meets orphan disease status (Orpha number: ORPHA102) (Wenning and Stefanova, 2009). Depending on the predominant motor presentation MSA is mainly classified into a parkinsonian variant (MSA-P) reflecting underlying striatonigral degeneration (SND) and a cerebellar variant (MSA-C) resulting from olivopontocerebellar atrophy (OPCA). As illustrated in Fig. 1, the degeneration of the MSA-P-, MSA-C- and autonomic failure-associated regions can appear in different combinations and different severities. There is no current evidence to suggest that pathogenesis differs in MSA-P and MSA-C based on overlapping neuropathologies including GCI deposition (Ubhi et al., 2011). MSA-P accounts for 60% of patients in the Western hemisphere whereas MSA-C appears to be predominant in East-Asian countries such as Japan (Watanabe et al., 2002, Wenning et al., 1994a). However, also other subtypes that do not fit to this classification have been described such as minimal-change MSA or MSA with disease duration that widely exceeds the usual survival time of 6–9 years after disease onset (Piao et al., 2001, Wenning et al., 1994b). In addition to progressive motor impairment, most MSA patients develop features of autonomic failure including orthostatic hypotension and urogenital disturbances such as increased frequency, enhanced urgency, incontinence and/or retention associated with male erectile dysfunction and female genital hyposensitivity. Especially in early disease stages, MSA-P might often be mistaken for PD and genetic or secondary late-onset ataxias may be labeled as MSA-C due to similarities in disease presentation (Gilman et al., 2008). A definite diagnosis requires the finding of oligodendrogliopathy at autopsy, which is characterized by alpha-synuclein (α-Syn)-positive glial cytoplasmic inclusions (GCIs, Papp-Lantos bodies) throughout the central nervous system (CNS), and the presence of neurodegenerative changes consistent with SND, OPCA and autonomic degeneration of predominantly central origin (Trojanowski and Revesz, 2007). Consensus diagnostic guidelines for possible, probable and definite MSA have been proposed and validated (Gilman et al., 1998, Gilman et al., 2008). So far there is no cure available for this disease and symptomatic therapies are limited (Wenning and Stefanova, 2009). Therefore there is a strong need to further investigate the mechanisms of neurodegeneration and find interventional treatment options. Here we review the progress over the last decade in defining pathogenetic targets of MSA and the effects of candidate neuroprotective and neurorestorative interventions utilizing the growing number of *in vitro* and *in vivo* MSA models. This work has led to several clinical trials and there is hope that an effective intervention could be identified in the near future.

## 2 Etiology

The etiology of this fatal disease remains to be further investigated, however, there is evidence that it is caused by a combination of genetic predisposition and environmental influences. Single nucleotide polymorphisms (SNPs) at the SNCA locus coding for α-Syn have been identified and patients with SNCA duplications and triplications have been found to manifest clinical and pathological features that are similar to those seen in MSA (Al-Chalabi et al., 2009, Fuchs et al., 2007, Scholz et al., 2009). Recently, Holton and colleagues reported a G51D mutation in the SNCA locus and described mixed pathological features of PD and MSA suggesting that investigation of this mutation could help in discovering the exact mechanisms of α-Syn malfunction (Kiely et al., 2013). However, the connection between the SNCA locus and MSA could not be confirmed in an independent genome wide association study (Sailer, 2012).

Genetic forms of MSA appear to be very rare (Hara et al., 2007, Wullner et al., 2004, Wullner et al., 2007). A recent study of autosomal recessive MSA families from Japan reports mutations in the COQ2 gene which encodes an enzyme essential for the biosynthesis of coenzyme Q_10_ and is thereby associated with causative mitochondrial dysfunction (The Multiple-System Atrophy Research Collaboration, 2013). Screening for COQ2 polymorphisms in sporadic MSA cases revealed variants that conferred increased disease risk for MSA in Japanese cohorts further linking dysfunctional COQ2 with MSA pathogenesis (The Multiple-System Atrophy Research Collaboration, 2013). However, the mutations were only found in a few family members of the multiplex families strongly suggesting that there are also other MSA genes that have not been identified yet (The Multiple-System Atrophy Research Collaboration, 2013). Many association studies have been performed in order to identify genes related to MSA pathology (extensively discussed in Stemberger et al., 2011b, Wenning and Stefanova, 2009).

Epidemiological data have shown that MSA patients reported more frequent exposure to environmental toxins and a history of farming than neurologically healthy controls. Similar to PD patients, non-smokers were less frequent among MSA patients compared to controls (Vanacore et al., 2000). Regular consumption of fish, aspirin or alcohol was more frequently recorded in healthy control subjects than patients, whereas lower education level and daily consumption of meat seemed more common in MSA (Vidal et al., 2008). Since MSA has a low prevalence, study sizes to identify possible environmental risk factors have been relatively small; therefore, results were at times inconclusive and further investigations will be required for validation.

## 3 Pathology

Pathologically, MSA is characterized by selective vulnerability of the central autonomic, striatonigral and olivopontocerebellar networks. Depending on the predominant motor presentation, SND or OPCA is the prevailing pathology (Ozawa et al., 2004). A dark-brown discoloration of the putamen due to lipofuscin, neuromelanin and increased iron pigment can often be found in MSA-P brains. The lesser response to l-dopa treatment (than seen in typical PD patients) likely reflects the striatal disease process with progressive loss of dopamine receptors and striatopallidal output systems (Ito et al., 1996, Wenning et al., 1994a). MSA-C, the cerebellar variant, is characterized by neurodegeneration in the inferior olives, pontine nuclei, paleocerebellum, neocerebellum, and middle cerebellar peduncles (Wenning et al., 1996b).

In addition, cell loss has been reported in autonomic brain stem nuclei underlying the characteristic autonomic features of MSA (Jellinger, 2011, Ubhi et al., 2011, Wakabayashi et al., 2010) (Fig. 1). Early autonomic failure (AF), as defined by symptoms reported and signs typically recorded prior to or shortly after motor onset, is a distinctive feature that helps discriminate MSA-P from PD and MSA-C from other sporadic late-onset ataxias. The degree of neuronal loss and gliosis does not always correlate with AF severity, however, these non-motor signs of MSA are of great diagnostic relevance since they often precede the motor signs (Jecmenica-Lukic et al., 2012). Preganglionic efferent autonomic dysfunction and impairment of neuronal networks in the brainstem are thought to be the main causes for the progressive autonomic symptoms associated with MSA (Kollensperger et al., 2007a, Ozawa, 2007). For example, orthostatic hypotension (OH) in MSA patients correlates with loss of preganglionic sympathetic neurons in the intermediolateral cell column (Benarroch, 2002). C1 catecholaminergic neurons and other neurons of the ventrolateral medulla are associated with maintenance of tonic sympathetic vasomotor tone and have been shown to be significantly depleted in MSA patients (Benarroch et al., 1998, Kato et al., 1995). Involvement of the dorsal motor nucleus of the vagus and the ventrolateral portion of the nucleus ambiguus and loss of arginine–vasopressin neurons in the hypothalamus and suprachiasmatic nucleus contribute to cardiovagal disturbances and a dysregulation of circadian rhythms (Benarroch et al., 1998, Benarroch et al., 2006, Kaufmann, 1998). Several studies have investigated cardiac sympathetic innervation using meta-iodobenzylguanidine (MIBG) uptake and related scintigraphy in various parkinsonian disorders (Raffel et al., 2006). Collectively, these and related positron emission tomography (PET) studies have shown that postganglionic cardiac sympathetic denervation occurs in PD and dementia with Lewy Bodies, *i.e.*, diseases featuring predominantly neuronal (rather than glial) α-Syn inclusions in sympathetic ganglia, whereas cardiac innervation appears to be preserved in most, but not all, MSA cases (Raffel et al., 2006). The loss of TH immunoreactive neurons in the paravertebral sympathetic ganglia, while commonly found in PD, was only present in 6 of 15 MSA patients and, when found, was rated as less pronounced (Orimo et al., 2008). These data suggest that cardiovascular AF in MSA results mainly from the degeneration of preganglionic neurons in the central portion of the autonomic nervous system. In contrast to PD, striatal dopaminergic denervation in MSA could not be correlated with cardiac denervation, thereby further suggesting that the responsible pathological processes in these two diseases are different (Raffel et al., 2006). Urogenital dysfunction plays a predominant and early role in MSA reflecting loss of preganglionic sympathetic and parasympathetic neurons of the sacral spinal cord (Ozawa, 2007). The frequently observed changes in respiratory frequency seen in MSA patients have been linked to depletion of serotonergic raphe neurons and NK-1R neurons of the ventrolateral medulla and A5 noradrenergic neurons in the pons (Benarroch, 2003).

Histopathologically, the following main characteristics of MSA have been described: (1) selective neuronal loss and axonal degeneration, (2) predominantly oligodendroglial α-Syn accumulation, (3) degenerating myelin and (4) astro- and microgliosis (Trojanowski and Revesz, 2007). GCIs, also called Papp-Lantos inclusions (Papp et al., 1989) have been recognized as the main morphological hallmark for definite neuropathological diagnosis of MSA and thought to play an important role in disease pathology (Jellinger and Lantos, 2010, Trojanowski and Revesz, 2007). In MSA, five types of α-Syn positive cellular inclusions have been reported including glial cytoplasmic inclusions (GCIs) and glial nuclear inclusions (GNIs) as well as accumulations in the nuclei and cytoplasm of neurons (NNIs and NCIs) (Duda et al., 2000a, Duda et al., 2000b, Papp et al., 1989). Additionally, astroglial cytoplasmic inclusions (ACIs) have been reported (Wenning and Jellinger, 2005). GCIs have been described as agyrophilic, half-moon- or sickle-shaped, triangular, oval or conical accumulations mainly composed of filamentous α-Syn (Tu et al., 1998). However, besides α-Syn as the main component, GCIs contain a variety of other proteins including ubiquitin, tau, p25-alpha (p25α), LRRK2, Parkin, prion disease-linked 14-3-3 protein, members of the heat shock protein (Hsp) 70 and heat shock cognate protein (Hsc) 70 family, α-tubulin, β-tubulin, microtubule-associated proteins, DARRP-32, and cyclin-dependent kinase 5, but their exact role in the misfolding and aggregation process still needs to be elucidated (Gai et al., 2003, Giasson et al., 2003, Huang et al., 2008, Kawamoto et al., 2007, Song et al., 2007). The number of GCIs has been found to be increased with longer disease duration and there is a positive correlation between the GCI density and neurodegeneration in various areas highlighting the fact that GCI formation likely plays an important role in MSA pathogenesis and suggesting that neuronal loss might be secondary to a prominent oligodendrogliopathy (Ozawa et al., 2004, Wenning et al., 2008).

Recently, it was shown that tau and α-Syn do not accumulate in oligodendrocyte precursor cells (OPCs) (Ahmed et al., 2013) and the density of OPCs was increased in a white matter region that is severely affected by GCI pathology and myelin degeneration. The authors claim that the increased numbers of OPCs most likely represent a disease-specific repair mechanism.

The highest GCI density in the gray matter has been reported in the deeper laminae of primary motor and premotor cortex, putamen, globus pallidus, subthalamus, SN, brainstem nuclei and the intermediolateral column of the spinal cord (Ozawa et al., 2004). The white matter areas that are most affected are the subcortical motor regions, internal and external capsule, corpus callosum, corticospinal tracts and the middle-cerebellar peduncle (Inoue et al., 1997, Ozawa et al., 2004, Tu et al., 1998).

NCIs occur less frequent than GCIs but are still widely abundant in the CNS.

The different types of neuronal inclusions have a similar distribution, affecting cortical, subcortical, brainstem, and cerebellar nuclei, being particularly prevalent in pontine basis and inferior olives (Nishie et al., 2004b).

## 4 Animal models

### 4.1 Neurotoxin MSA models

Neurotoxin models mimic the refractory DA responsiveness of MSA-P and they replicate SND, the neuropathology underlying parkinsonism associated with the human disorder. However, these MSA-P models do not replicate GCI pathology. They have primarily served as testbeds for characterizing the effects of neurorestorative interventions. In addition, several neuroprotection studies have also been performed in MSA toxin models. The different mouse and rat neurotoxin approaches replicate different degrees of damage and can therefore be used to mimic early or advanced disease stages.

The first MSA models were developed using stereotaxic administration of striatonigral neurotoxins in rats to cause the neuronal loss characteristic of SND underlying MSA-P. An early approach using a single-toxin to cause lesions in the rat striatum and retrograde damage of the dopaminergic system in the SN involved 1-methyl-4-phenylpyridinium (MPP+) (Storey et al., 1992). Stereotaxic injection of 3-nitropropionic acid (3-NP) was initially applied to reproduce animal models of Huntington's disease (HD) and has been used in MSA rat models to cause a significant loss of striatal neurons, reduction of the striatal surface area and retrograde loss of dopaminergic neurons in the substantia nigra (Borlongan et al., 1995, Waldner et al., 2001). Also 6-hydroxydopamine (6-OHDA), which has initially been used to create animal models of PD, and quinolinic acid (QA), previously used to generate HD models, have been used in rats to induce unilateral SND with neuronal loss in the substantia nigra and striatum (Ghorayeb et al., 2001, Kollensperger et al., 2007b, Kollensperger et al., 2009, Puschban et al., 2000a, Puschban et al., 2000b, Scherfler et al., 2000, Venero et al., 1995, Wenning et al., 1996a). Following this earlier work, different application protocols of 6-OHDA into the medial forebrain bundle or striatum, simultaneously or sequentially applied with QA, have been established in rats to create milder degrees of SND lesions aiming to optimize the outcome of neurorestorative interventions (Kaindlstorfer et al., 2012, Kollensperger et al., 2009).

Systemically administered neurotoxins to induce mouse models of SND include 1-methyl-4-phenyl-1,2,3,6-tetrahydropyridin (MPTP) followed by 3-NP, 3-NP followed by MPTP as well as simultaneous administration of 3-NP and MPTP (Diguet et al., 2005, Fernagut et al., 2002, Fernagut et al., 2004, Stefanova et al., 2003). The preceding dopaminergic depletion caused by MPTP appears to have a protective effect on the striatal neurons in the sequential models, whereas MPTP potentiates the effect of 3-NP in the simultaneous administration model. 3-NP has also been used in transgenic models to induce MSA-like pathology by generating oxidative stress (Stefanova et al., 2005, Ubhi et al., 2009).

Before the involvement of α-Syn in MSA was known, the toxin models served as testbeds for lesion studies with transplantation approaches. However, none of these toxin models displayed the characteristic GCI pathology, which is a severe pitfall since it has been shown that transplantation into pro-inflammatory MSA environment results in decreased graft survival (Stefanova et al., 2009b). Thereby, the results from transplantation studies in purely toxin-induced lesion models without presence of α-Syn-positive aggregates might not hold true for studies in brains with MSA pathology.

### 4.2 Transgenic MSA models

Since the discovery of α-Syn as a key player for MSA and the development of transgenic models there has been a shift towards translational pathogenesis research and the role of α-Syn in MSA. The transgenic MSA mouse models mostly depend on the overexpression of human α-Syn (hα-Syn) under the control of different oligodendroglial promoters (PLP (Kahle et al., 2002), MBP (Shults et al., 2005), CNP (Yazawa et al., 2005)) (see Table 1 for an overview of these transgenic models).

**Table 1:** Overview of characteristic features in human MSA compared to the transgenic models.

	Human	PLP-α-Syn mouse	CNP-α-Syn mouse	MBP-α-Syn mouse
Glial pathology				
GCI formation	✓	✓	✓	✓
Microgliosis	✓	✓	n.r.	n.r.
Astrogliosis	✓	✓	✓	✓
Neuronal pathology				
SND	✓	Degeneration of SNpc and striatum	n.r.	Degeneration of dopaminergic nerve terminals in the striatum
OPCA	✓	OPCA-like pathology only after 3-NP challenge	n.r.	Neuropathological alterations in the cerebellum
Central autonomic degeneration	✓	✓	n.r.	n.r.
Motor impairment	✓	✓	✓	✓
Non-motor features				
Urological disturbances	✓	✓	n.r.	n.r.
Cardiovascular disturbances	✓	✓	n.r.	n.r.
Anhidrosis	✓	n.r.	n.r.	n.r.
Respiratory disturbances	✓	n.r.	n.r.	n.r.
Olfactory disturbances	–	–	n.r.	✓
References	Benarroch, 2002, Jecmenica-Lukic et al., 2012, Stefanova et al., 2009a, Wenning and Stefanova, 2009, Wenning et al., 2013	Boudes et al., 2013, Kahle et al., 2002, Krismer et al., 2013, Kuzdas et al., 2013, Stefanova et al., 2005, 2008, 2009b, 2012, Stemberger et al. (2010, 2011a) (Flabeau et al., 2014)	Nakayama et al., 2009, Nakayama et al., 2012, Yazawa et al., 2005	Shults et al., 2005, Ubhi et al., 2008, Ubhi et al., 2009, Ubhi et al., 2010b, Ubhi et al., 2012

✓, present; –, not present; n.r., not reported.

Kahle et al., 2002 created a mouse model using the myelin proteolipid protein (PLP) promoter, constitutively overexpressing α-Syn in oligodendrocytes and were able to detect insoluble triangular or half-moon-shaped α-Syn inclusions arranged around the nucleus of oligodendrocytes. Another important feature of this transgenic animal model is the hyperphosphorylation at the Ser129 residue of full-length α-Syn, which is also present in human brain tissue and leads to the pathologic insolubility of the overexpressed protein in the cytosol of oligodendrocytes (Kahle et al., 2002). An important finding originally described by Stefanova et al., 2007 analyzing this model reports higher striatonigral levels of activated microglia in adult transgenic mice compared to wildtype (wt) controls, suggesting that the innate immune system and microglial activation may play a role in neurodegenerative pathways of MSA. The PLP-α-Syn mouse model replicates early MSA stages with mild but robust locomotor deficits (Stefanova et al., 2005) as well as pre-motor features including reduced heart rate variability (Kuzdas et al., 2013) and neurogenic bladder dysfunction (Boudes et al., 2013).

PLP-α-Syn mice do not show any overt demyelinating pattern but instead feature a mild degree of multisystem neuronal degeneration involving motor and autonomic nuclei such as the substantia nigra pars compacta (SNpc), locus coeruleus (LC), laterodorsal tegmental nucleus (LDT), pedunculopontine tegmental nucleus (PPT), nucleus ambiguus (NA) and Onuf's nucleus (Stefanova et al., 2005, Stemberger et al., 2010).

Masliah and colleagues generated a more aggressive transgenic model that depends on the myelin basic protein (MBP) promoter (Shults et al., 2005). The rapidly progressive phenotype in this model involves basal ganglia, neocortex and cerebellum with no marked degeneration of the dopaminergic SN neurons. The neuropathology includes degenerative changes and myelin alterations consistent with motor deficits that increase over time and mitochondrial alterations (Shults et al., 2005, Ubhi et al., 2009). Recently, the authors found upregulation of the micro-RNA miR-96 and claim that dysregulation of miR-96 could play a role in the pathogenesis of MSA (Ubhi et al., 2013).

Another model using the 2′,3′-cyclic nucleotide 3′-phosphodiesterase (CNP) promoter to induce α-Syn overexpression in oligodendrocytes has been created by Yazawa et al., 2005. They could detect hα-Syn aggregates in oligodendrocytes as well as extensive degeneration of the spinal cord motor neurons and pyramidal tracts reflecting axonal accumulation of endogenous mouse α-Syn. Furthermore the authors report significant neuronal and oligodendroglial loss in aged animals compared to non-transgenic controls as well as demyelination and severe levels of gliosis in the brain and spinal cord. The behavioral analysis showed reduced grip strength and decreasing performances in the rotarod motor examination, starting at an average age of seven to nine months, reflecting progressive neurodegeneration.

Since these α-Syn overexpressing mouse models have utilized different promoters, they might affect distinct subpopulations of oligodendrocytes leading to phenotypic heterogeneity.

Another model that presents MSA-like cardiovascular autonomic failure and characteristic neuronal loss and additional oligodendroglial α-Syn aggregations works with the overexpression of the α1_B_-adrenergic receptor (α1_B_-AR) (Zuscik et al., 2000). Perez and colleagues analyzed the effect of long-term treatment with an α1-AR-antagonist and were able to prevent neurodegeneration and the formation of α-Syn aggregates (Papay et al., 2002). However, the link between the α1_B_-AR and MSA pathology is still unclear and this animal model exhibits atypical features like epileptic seizures (Kunieda et al., 2002).

Since the discovery of α-Syn in GCIs the genetic application of hα-Syn has been used as a valuable tool to investigate potential disease-modifying treatments that aim to interfere with protein aggregation and thereby prevent neurodegeneration. However, it is far from optimal since most of these animal models only display mild signs of neurodegeneration. Furthermore, introducing a human protein into a murine organism might have species-specific effects. Some of the tested drugs were found to have beneficial effects in animal models, however, in some studies the treatment was started before disease induction by systemic application of 3-NP. In addition, there is a problem with finding the optimal dose since the efficient doses used in mice might not be applicable in humans due to safety concerns or side-effects (see below). Different therapeutic approaches have been taken to explore various targets associated with MSA pathogenesis, yet, an effective treatment still has to be discovered.

## 5 Pathogenesis

**Fig. 2:**
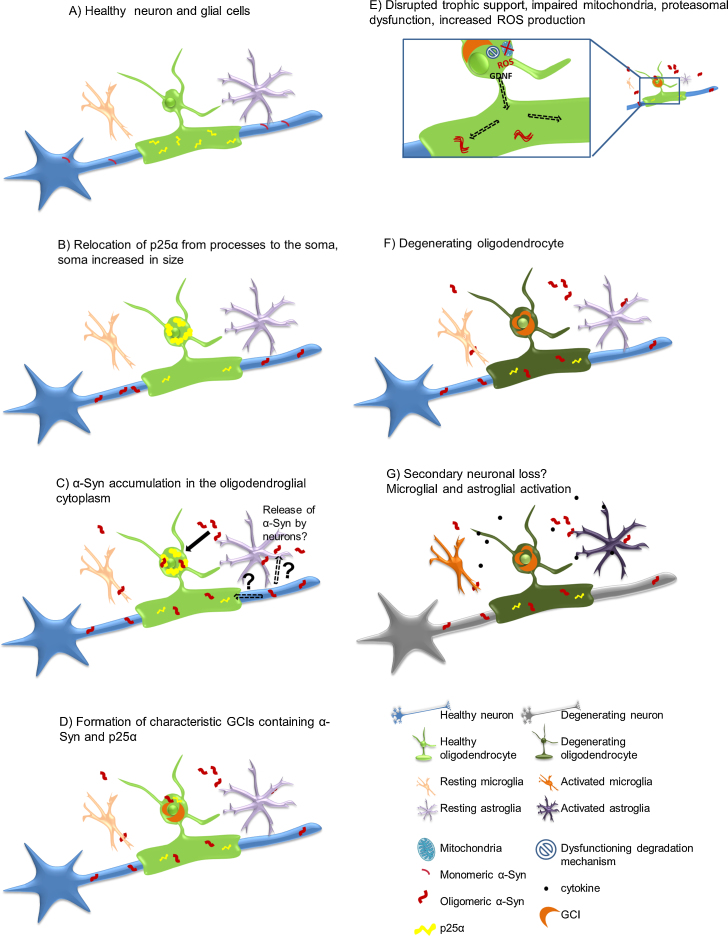
Possible pathological α-Syn-spreading and accumulation mechanism leading to neurodegeneration. (A) Healthy neuron, oligodendrocyte, microglia and astrocyte, p25α mainly located in the myelinating oligodendroglial processes, monomeric α-Syn present in presynaptic nerve terminals. (B) Relocalisation of p25α from the processes to the soma, inclusion formation and swelling of the oligodendroglial soma. (C) Oligomeric α-Syn accumulation in the oligodendroglial cytoplasm, the exact source of α-Syn remains to be investigated. Possible hypotheses include exocytosed α-Syn from neurons and uptake into oligodendrocytes by cell-to-cell propagation or upregulation of α-Syn expression in oligodendrocytes themselves. In addition, axonal α-Syn may be taken up by the dysfunctional oligodendroglial myelin compartment. (D) α-Syn aggregates form insoluble half-moon shaped GCIs characteristic for the disease. (E) Disruption of trophic support (*e.g.* GDNF), mitochondrial failure, increased production of reactive oxygen species (ROS) and proteasomal dysfunction occur. (F) Oligodendrocytes suffer from severe distress and will eventually degrade. (G) Activation of micro/astroglial cells by cytokines released from the damaged oligodendrocytes, proposed secondary neuronal loss potentially due to lack of trophic support, ROS production, proteasomal failure and pro-inflammatory environment.

An increasing number of studies has been conducted in the past years aiming to unravel the pathogenic mechanism in MSA, mainly focusing on the event of α-Syn-misfolding and GCI-formation since the widespread appearance of GCIs with fibrillar α-Syn as their main component is a hallmark of MSA pathology (Ozawa et al., 2004, Papp et al., 1989, Papp and Lantos, 1994, Spillantini et al., 1998, Trojanowski and Revesz, 2007). In addition to aggregation of misfolded α-Syn in oligodendrocytes, oxidative stress resulting from mitochondrial dysfunction, excitotoxicity, neuroinflammation and metabolic and gene expression changes may be important factors in the pathogenesis of MSA (Fig. 2).

### 5.1 α-Syn in MSA

α-Syn, a heat stable protein of 140 amino acids, is mainly located at presynaptic nerve terminals and thought to have a role in synaptic maintenance and formation of the soluble N-ethylmaleimide-sensitive factor attachment protein receptor (SNARE) complex which is essential for the formation of synaptic vesicles and neurotransmitter release (Burre et al., 2013, Murphy et al., 2000). The reason for progressive α-Syn accumulation in MSA oligodendroglia remains entirely unclear.

Converging evidence suggests that MSA is a unique α-synucleinopathy with oligodendroglial inclusion pathology and secondary neuronal loss (Wenning et al., 2008). It is not clear whether the intracellular α-Syn steady-state is increased due to a pathological upregulation within oligodendrocytes, which to date has only been shown in *ex vivo* experiments, or if the expression level of *SNCA* is normal yet its uptake from CSF is markedly enhanced as suggested by recent CSF quantification studies (Mollenhauer et al., 2011, Mollenhauer et al., 2012). Alternatively, the primary pathological event could be linked to a dysfunction in the degradation of α-Syn within affected oligodendroglia (Burn and Jaros, 2001, Miller et al., 2005, Mollenhauer et al., 2011). Recent studies suggested that oligomeric and phosphorylated fragments of α-Syn may be relevant in the pathogenesis of MSA and other synucleinopathies, therefore lending support to efforts that seek to prevent the formation of α-Syn oligomers and reduce the degree of phosphorylation (Foulds et al., 2012, Wang et al., 2012, Winner et al., 2011). Several studies aimed to evaluate the potential of α-Syn as a biomarker leading to contradictory results some confirming an altered ratio of total to phosphorylated α-Syn as a valid biomarker (Foulds et al., 2012, Wang et al., 2012), and others reporting no significant differences between CSF samples of MSA patients and PD, PSP or DLB patients or healthy controls (Aerts et al., 2012, Mollenhauer et al., 2011, Shi et al., 2011). It is still unclear where the α-Syn that forms the insoluble aggregates in oligodendrocytes, originally comes from. Comparisons of brain mRNA levels between MSA patients and healthy controls did not reveal any differences (Jin et al., 2008, Miller et al., 2005, Ozawa et al., 2001). Since α-Syn is a neuronal protein, it may originate from dysfunctional neurons, however, why neurons eject the protein, whether that is due to an excessive accumulation at the pre-synaptic site, and if so, what causes this overload, is still unclear.

Since dopamine-rich grafts in PD patients were immunoreactive for α-Syn inclusions years after transplantation, and the fact that α-Syn is not natively expressed in oligodendrocytes (Miller et al., 2005, Ozawa et al., 2001), there is an intense debate regarding the spread of pathologically folded α-Syn species in PD and related disorders such as MSA between cells and cell types (Fellner et al., 2011, Fellner and Stefanova, 2012, Kordower et al., 2008, Lee et al., 2011, Steiner et al., 2011). Neurons probably release excessive α-Syn and neighboring cells endocytose it from the surrounding medium/extracellular space (Lee et al., 2010c) (Fig. 2). *Ex vivo* and *in vivo* experiments showed that direct neuron-to-neuron and neuron-to-astroglia transmission of α-Syn can indeed occur in PD models (Desplats et al., 2009, Lee et al., 2010a, Luk et al., 2012). There were a number of reports lately focusing on the spreading mechanism of pathologically folded α-Syn from cell-to-cell and even between different cell types. It has been shown that α-Syn is transferred from α-Syn overexpressing host cells in a transgenic PD model to the grafted embryonic mesencephalic neurons from wt mice (Hansen et al., 2011). After showing that neurons and other brain cells can take up extracellular α-Syn aggregates (Lee et al., 2008a) and confirming the uptake of extracellular α-Syn by mouse cortical neuronal stem cells, they analyzed whether mouse cortical neuronal stem cells would take up α-Syn if transplanted into PD mice expressing human α-Syn under control of the neuronal Thy-1 promoter. Indeed, they found α-Syn aggregates in the grafted cells and report increased spreading with time (2.5% of grafted cells after one week, 15% after four weeks) (Desplats et al., 2009). They further claim that the quality control system in the acceptor cells must be impaired and therefore promote the accumulation of transmitted α-Syn. Last, they also report signs of cell fragmentation and apoptosis such as caspase-3-activation in cells that have taken up extracellular α-Syn in *in vivo* and *in vitro* experiments. One year later, the same group published results confirming neuron-to-astroglia transfer of α-Syn by endocytosis and the formation of inclusion bodies again in cell culture as well as transplantation studies (Lee et al., 2010a). They also found that astrocytes that were exposed to neuronal α-Syn displayed altered gene expression reflecting an inflammatory response and increased cytokine and chemokine levels which correlated with the extent of astroglial α-Syn uptake (Lee et al., 2010a). Another interesting study has been published recently, injecting synthetic α-Syn-fibrils unilaterally into healthy mice (Luk et al., 2012). They report transmission of α-Syn throughout the established neuronal networks with increased propagation over time on the ipsilateral and even to the contralateral side. Furthermore, the spreading of α-Syn in healthy mice led to a decrease of dopaminergic cells on the ipsilateral side and a significant worsening in behavioral motor ability assessment (Luk et al., 2012). Another recent study tested the effects of brain homogenates from confirmed MSA cases or a spontaneously ill homozygous transgenic PD mouse line overexpressing the A53T mutation of α-Syn injected into a heterozygote A53T mouse line with luciferase reporter system under control of the GFAP promoter (Watts et al., 2013). They found that brain homogenates of MSA patients or the PD mouse line led to insoluble α-Syn-aggregates in the brains of recipient mice following unilateral inoculation supporting the prion-like spreading hypothesis. However, oligodendroglial aggregates of phosphorylated α-Syn were not reported in this study. α-Syn-immunoreactive cells have been found within the graft area three months after transplantation in the transgenic PLP-α-Syn mouse model, however, these cells were of host origin (Stefanova et al., 2009b). To this date, there is little proof of a direct transfer of α-Syn to oligodendrocytes, however, a recent study that utilized cross-breeding a mouse line with neuronal α-Syn-expression with one that has primary oligodendroglial expression of α-Syn, demonstrated that the double-transgenic offspring more closely resembled the oligodendroglial α-Syn-expressing line (Rockenstein et al., 2012). Instead of a mixed and equal distribution of hα-Syn in neurons and oligodendrocytes, the authors found a larger proportion in oligodendrocytes for which they suggest three possible explanations: (1) translocation of neuronal α-Syn to oligodendrocytes, (2) downregulation of neuronal α-Syn caused by unidentified signals from the oligodendroglial cells, and (3) clearance of neuronal α-Syn. Together, these findings support the theory of cell-to-cell transmission of (pathologically folded) α-Syn and would offer a potential mechanism of how the neuronal protein α-Syn is transferred to oligodendrocytes in MSA. However, it should be emphasized that the exact mechanisms underlying this prion-like transfer and the relative contribution of soluble and oligomeric species of α-Syn as well as of other pathologic proteins in this propagation require further elucidation (Angot et al., 2010, Angot et al., 2012, Frost and Diamond, 2010, Hansen et al., 2011, Jellinger, 2012, Lee et al., 2010c, Steiner et al., 2011, Walker et al., 2013).

### 5.2 Potential factors involved in α-Syn-aggregation

Even though it is unclear where the α-Syn that aggregates in GCIs originates from, and how oligodendroglial α-Syn causes neurodegeneration, the important role of oligodendroglial pathology in MSA is strengthened by the finding that p25α accumulates very early in MSA pathogenesis (Song et al., 2007). P25α is a phosphoprotein specific for oligodendrocytes which is also known as tubulin polymerization-promoting protein (TPPP) and involved in myelination and stabilization of microtubules (Ovadi and Orosz, 2009, Takahashi et al., 1991). It has been shown that in MSA patients, the location of p25α is shifted from the myelin sheath towards the oligodendroglial cell bodies (Song et al., 2007). Changes in myelin integrity have been identified in MSA patients and connected with major changes in p25α and myelin basic protein (MBP) (Matsuo et al., 1998, Song et al., 2007). It has been shown that the presence of p25α promotes aggregation of α-Syn *in vitro* (Lindersson et al., 2005) and a large proportion of oligodendrocytes show abnormal distribution patterns of p25α which often co-localize with insoluble α-Syn-aggregates (Song et al., 2007, Wenning et al., 2008). Phosphorylation of α-Syn at the Ser129 locus and a shift of the ratio of total to phosphorylated α-Syn have been shown to be involved in the aggregation processes of PD and other synucleinopathies (Kragh et al., 2009, Nishie et al., 2004a, Wang et al., 2012), however, the exact role of the Ser129 phosphorylation in the aggregation process remains unclear. In PD models it has actually been shown that the phosphorylation at the Ser129 locus might occur after aggregation of α-Syn (Sato et al., 2013). Studies report that in rat oligodendrocytes which neither expressed endogenous α-Syn, nor p25α, the coexpression of those two factors led to MSA-like degeneration and phosphorylation of α-Syn at the Ser129 locus further highlighting the involvement of p25α (Kragh et al., 2009).

Furthermore, almost 50% of oligodendrocytes display abnormal accumulation pattern of p25α in α-synucleinopathies (Lindersson et al., 2005). This, and the fact that p25α is associated with the function of the microtubular system strengthen the hypothesis that p25α might play a role in the pathogenic aggregation process of α-Syn in MSA.

Together, the finding of p25α and MBP relocation and alterations support the hypothesis of MSA being a primary oligodendrogliopathy followed by secondary, selective neurodegeneration (Wenning et al., 2008).

Nakayama et al., 2009 analyzed potential interaction partners of α-Syn in the CNP α-Syn mouse model of MSA, focusing especially on proteins involved in protein aggregation. They found that the presence of oligodendroglial hα-Syn leads to the accumulation of endogenous mouse α-Syn and further induces secondary axonal aggregation of α-Syn which causes neurodegeneration (Yazawa et al., 2005) and identified beta-III tubulin as a potential key player in the pathological disease mechanisms (Nakayama et al., 2009). This is consistent with the detection of beta-III tubulin in GCIs of human MSA patients (Abe et al., 1992, Nakazato et al., 1990, Tu et al., 1998). Further evidence that the interaction between α-Syn and beta-III tubulin is an important step in the GCI formation, can be seen when applying microtubule-depolymerizing agents, which prevent the formation of the insoluble aggregates (Nakayama et al., 2009). Since α-Syn is believed to be a regulator of synaptic vesicle function (Gitler and Shorter, 2007), a decreased number of synaptic vesicle fusion events have been detected in this transgenic MSA model (Yazawa et al., 2005), which could be an important cause for neuronal degeneration. These findings are of great importance for developing therapeutic strategies interfering with these disease mechanisms.

Autophagy is an essential degradation process important for selective disposal of misfolded protein aggregates and damaged organelles and thereby reduces the risk for development of neurodegenerative diseases that involve aggregation of misfolded proteins (Lee et al., 2010b). Histone deacetylase-6 (HDAC6) is a key-player for the fusion of autophagosomes with lysosomes and deficiency leads to impaired maturation of the autophagosome and increased accumulation of aggregated protein.

Recent work has shown that GCIs are also immunoreactive for histone deacetylase (HDAC) 6 antibodies indicating impaired HDAC6 function, thus providing a link to autophagy dysfunction (Miki et al., 2011). Findings from cell culture experiments report increased α-Syn levels after treatment with HDAC6 inhibitor and also 3-methyladenine, an autophagy inhibitor, providing evidence that the disruption of the autophagocytic pathway may be involved in the pathogenesis of α-synucleinopathies (Su et al., 2011).

### 5.3 Alterations in cell death regulation and trophic support

There are reports of increased expression of FAS, a plasma membrane death receptor, in patients of neurodegenerative diseases (Choi et al., 1999, Hovelmeyer et al., 2005, Kragh et al., 2013). In primary oligodendroglial cell culture from the PLP-α-Syn mouse model, it has recently been shown that the expression of FAS was increased early in GCI formation, suggesting that this α-Syn-dependent enhanced sensitivity to FAS signaling might be involved in the pathogenic mechanisms and therefore serve as a potential target for disease modifying treatment strategies (Kragh et al., 2013).

A key question in MSA research has been how α-Syn accumulation, predominantly in oligodendrocytes, can lead to death of another cell type, namely neurons. Oligodendrocytes have many roles in supporting neuronal function, the most notable being myelination. Oligodendrocytes express neurotrophic factors, including glial-derived neurotrophic factor (GDNF) (Wilkins et al., 2003), brain-derived neurotrophic factor (BDNF), and insulin-like growth factor 1 (IGF-1) (Wilkins et al., 2001) that are involved in the maintenance and survival of neuronal populations. Correlating to reports in humans, GDNF levels were also reduced in the MBP-α-Syn mouse model of MSA and motor deficits could be reduced with intracerebroventricular infusion of the neurotrophic factor (Ubhi et al., 2010b). One possible explanation for how oligodendroglial accumulation of α-Syn may result in neuronal death is that altered communication between neurons and oligodendrocytes, perhaps due to perturbation of this neurotrophic support, may contribute to neurodegeneration (Ubhi et al., 2011).

### 5.4 Mitochondrial dysfunction and oxidative stress

Many synucleinopathies are thought to affect mitochondrial function (Akundi et al., 2011, Lin and Beal, 2006, McCoy and Cookson, 2011, Xie et al., 2010), and pesticide exposure which is known to affect mitochondrial function has been linked to increased incidence of MSA (Hanna et al., 1999). The importance of oxidative stress (OS) in MSA has been demonstrated by post-translational modifications of α-Syn, including nitration, oxidation or phosphorylation at Ser129, and evidence from *in vitro* and *in vivo* MSA models (Duda et al., 2000a, Giasson et al., 2000, Kahle et al., 2002, Nishie et al., 2004a, Stefanova et al., 2005, Ubhi et al., 2009). During OS conditions, levels of reactive oxygen species (ROS) like oxygen (O_2_)-derived free radicals, hydrogen peroxide (H_2_O_2_) or hydroxyl (OH^−^) radicals are increased and/or cellular antioxidant protection-mechanisms can be impaired, which makes the cells increasingly vulnerable to OS and leads to protein, lipid and DNA damage.

Administration of the mitochondrial toxin 3-NP exacerbated behavioral deficits in PLP-α-Syn transgenic mice by inhibition of the mitochondrial chain and was associated with MSA-like neurodegeneration and enhanced dopaminergic cell loss reflecting the full-blown MSA pathology (Stefanova et al., 2005). Administration of 3-NP in the MBP-α-Syn transgenic mouse model also resulted in widespread neuronal degeneration associated with altered levels of nitrated and oxidized α-Syn, but not affecting global levels of α-Syn (Ubhi et al., 2009). It has been shown that the toxic effects of 3-NP were dependent on the presence of α-Syn, because α-Syn knockout (KO) mice, although susceptible to 3-NP-induced OS, displayed reduced neuronal loss and dendritic pathology in comparison to 3-NP-treated MBP-α-Syn transgenic mice (Ubhi et al., 2010a). 3-NP did not have severe effects on motor abilities, degeneration of dopamine transporter and striatum in α-Syn KO mice, highlighting the hypothesis, that the deficits in MSA are specifically caused by α-Syn related mechanisms (Ubhi et al., 2010a). These combination models featuring transgenic overexpression of hα-Syn, combined with the 3-NP paradigm are the first models of MSA that replicate the likely interaction of genetic and environmental factors underlying the human disorder (Stefanova et al., 2005, Ubhi et al., 2009).

Besides causative COQ2 mutations reviewed above (The Multiple-System Atrophy Research Collaboration, 2013) significant expression changes for other genes associated with mitochondrial function, ubiquitin-proteasome function and/or protein modification have been reported (Langerveld et al., 2007, Soma et al., 2008).

One very prominent example for intracellular antioxidant defense is the NF-E2-related factor 2 (Nrf2), a transcription factor that is involved in phase-II detoxification and antioxidant enzymes (Johnson et al., 2002). Furthermore, Nrf2 has been shown to be involved in the modulation of the innate immune system (Rojo et al., 2010). Interestingly, microglial and astroglial activation due to pro-inflammatory molecules and inducible nitric oxide synthase (iNOS) have been implicated with neurodegenerative processes in MSA (Stefanova et al., 2007) and Nrf2 deficiency in combination with the presence of α-Syn has been shown to cause increased neuronal death and inflammation in early-stage PD (Lastres-Becker et al., 2012).

### 5.5 Neuroinflammation in MSA

Micro- and astroglia have an important role for brain homeostasis, however, excessive glial activation as known from many neurodegenerative diseases can also have damaging effects (Lull and Block, 2010, Luo and Chen, 2012, Pizza et al., 2011, Streit et al., 2004). Whether these inflammatory cascades are directly involved in the initial pathogenic events or represent a downstream effect due to the presence of α-Syn is still under debate. It has been shown, that post-translationally nitrated α-Syn directly activates microglia *via* a classical pathway (Reynolds et al., 2008, Su et al., 2008) and leads to an increase of pro-inflammatory molecules such as interleukins, tumor necrosis factor-α (TNF-α), or interferon-γ (Bartels et al., 2010, Su et al., 2009). The specific structure of misfolded α-Syn appears to be important for induction of this pro-inflammatory pathway (Beraud et al., 2013). The degree of microglial activation in MSA brains appears to be greater in areas with mild to moderate degeneration as compared to areas with more severe damage (Wakabayashi et al., 1998). Activation of the innate immune system has been reported in the PLP-α-Syn mouse model of MSA and suppression of microglial activation by early administration of minocycline protected dopaminergic neurons in the SN (Stefanova et al., 2007). More recent evidence suggests a dual role of microglial activation in MSA. It has been shown that Toll-like receptor 4 is required for the α-Syn-dependent activation of micro- and astroglia that is associated with the release of pro-inflammatory cytokines (Fellner et al., 2013). Furthermore impaired clearance of α-Syn due to Toll-like-receptor 4 deficiency accelerated neurodegeneration (Stefanova et al., 2011). In general, the term “activation” does not adequately describe the complex morphological and functional change microglia undergo when reacting to changes in the microenvironment (Colton and Wilcock, 2010, Harry and Kraft, 2012).

Converging evidence for the role of neuroinflammation in MSA comes from recent findings showing an upregulation of myeloperoxidase, a marker of neuroinflammation, in transgenic PLP-α-Syn mouse and human MSA brains. A specific inhibitor led to amelioration of motor behavior, neuroprotection, and suppressed microglial activation in the MSA mouse model (Stefanova et al., 2012).

### 5.6 Working hypothesis for the pathogenesis of MSA

Under normal conditions, oligodendrocyte precursor cells mature and myelinate axons, with microglia being in a quiescent state. p25α is located in the myelinating processes of oligodendrocytes and α-Syn in synapses and axons (Fig. 2A). The earliest stages of MSA pathogenesis are currently unknown but are likely to involve a relocation of p25α from the myelin sheaths to the oligodendroglial soma, which is associated with myelin dysfunction and an increase in cell soma size (Song et al., 2007) (Fig. 2B). This process appears to result in aberrant α-Syn accumulation in the oligodendroglial cytosol leading to activation of microglia, their processes becoming less ramified and thicker (Klegeris et al., 2008, Wenning et al., 2008). The source of oligodendroglial α-Syn remains unknown. Adult oligodendroglia normally do not express α-Syn and MSA postmortem studies failed to show oligodendroglial α-Syn mRNA upregulation (Miller et al., 2005, Ozawa et al., 2001). Therefore uptake from neuronally released α-Syn species by diseased oligodendroglia is a possibility (Rockenstein et al., 2012). The aberrant α-Syn in oligodendrocytes undergoes fibril formation (Riedel et al., 2009) and then aggregates to form GCIs (Kahle et al., 2002) (Fig. 2C). This process is enhanced by the misplaced p25α (Hasegawa et al., 2010), which is incorporated into inclusions even prior to α-Syn (Kovacs et al., 2004) (Fig. 2B–D). At this stage the oligodendrocyte is further enlarged and myelin degeneration may leave axons vulnerable to damage by pro-inflammatory molecules derived from activated microglia (Stefanova et al., 2007). The presence of GCIs leads to disorders of cellular function and death of oligodendrocytes (Hasegawa et al., 2010, Probst-Cousin et al., 1998). This could contribute to reduced oligodendroglial trophic support (Ubhi et al., 2010b) (Fig. 2E), chronic neuroinflammation (Streit et al., 2004), and ultimately, neurodegeneration (Fig. 2F and G). Such conditions in one or more areas of the brain could spread to other functionally connected networks, resulting in a system-specific pattern of neurodegeneration (Brundin et al., 2008). However, the exact mechanism of neurodegeneration has not been fully identified at this stage (Stefanova et al., 2009a). Furthermore, a prion-like spreading hypothesis for α-Syn has come up following transplantation studies in PD and recently, pre-clinical evidence is accumulating for the seeding and spreading mechanism in PD and MSA (Luk et al., 2012, Watts et al., 2013).

## 6 Disease modification strategies

### 6.1 Drug and other non-cell based interventions

**Fig. 3:**
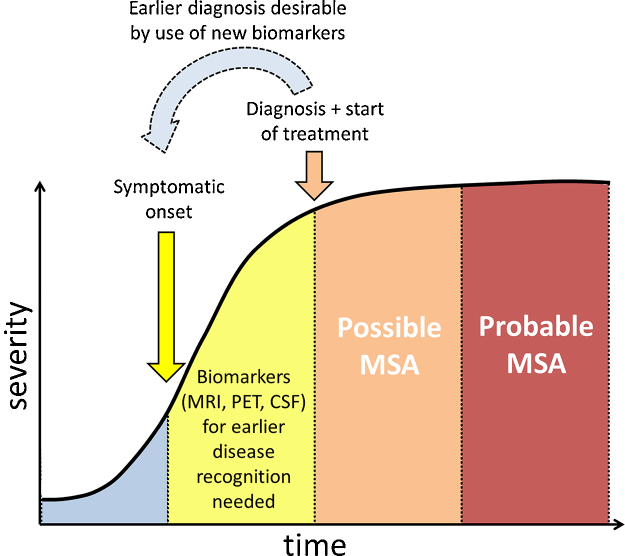
Biomarker-supported early diagnosis of MSA: a pre-requisite for successful trial intervention.

Neuroprotection is generally perceived as a therapeutic approach to attenuate or prevent neuronal degeneration, and thereby slowing or even forestalling disease progression (Shoulson, 1998). Since the pathogenesis of MSA is dominated by toxic α-Syn aggregation, optimal interventional strategies would need to prevent the formation of oligomers and/or clear already formed aggregates. Unfortunately, most clinical intervention trials focused on targets with an unclear relation to primary α-Syn aggregation and all drug or non-cell based trials have been negative so far likely reflecting multiple translational difficulties including lacking evidence of target engagement, underpowering, advanced disease stages at trial entry and lack of validated biomarkers (see Table 2, Table 3 for an overview of preclinical and clinical MSA studies and Fig. 3).

**Table 2:** Preclinical trials in MSA models.

Intervention	Animal model	Results
Riluzole (anti-glutamatergic drug)	Sequential double-toxin, double-lesion rat model (Scherfler et al., 2005)	Reduction of motor disturbances, reduction of the striatal lesion volume in the riluzole treated group compared to controls
	MPTP + 3-NP mouse model (Diguet et al., 2005)	Riluzole improved motor scores and decreased neurodegeneration of striatal neurons
Minocycline (tetracycline derivative)	Double-toxin, double-lesion rat model of MSA (Stefanova et al., 2004)	No behavioral effects, no neuronal protection, reduced microglial and astroglial activation
	PLP-α-Syn mouse model of MSA (Stefanova et al., 2007)	Significant reduction of neurodegeneration in the SNpc and striatum
Rasagiline (irreversible MAO-B inhibitor)	PLP-α-Syn mouse model of MSA combined with 3-NP administration (Stefanova et al., 2008)	Behavioral effects, relative preservation of olivopontocerebellar and striatonigral pathways
Rifampicin (antibiotic)	MBP-α-Syn mouse model (Shults et al., 2005, Ubhi et al., 2008)	Reduction of α-Syn aggregation
Nocodazole (microtubule-depolymerizing agent)	CNP-α-Syn mouse model of MSA (Nakayama et al., 2009)	Identified β-III-tubulin as key factor in α-Syn-aggregation, administration of nocodazole inhibited the aggregation of soluble α-Syn fibrils, but did not dissolve already formed aggregates
Terazosin (α1-AR antagonist)	α1B-Adrenergic receptor overexpressing transgenic MSA mouse model (Papay et al., 2002, Zuscik et al., 2000)	Long-term treatment improved motor deficits and reduced α-Syn-aggregation
Myeloperoxidase inhibitor (MPO)	PLP-α-Syn mouse model of MSA combined with 3-NP administration (Stefanova et al., 2012)	Reduced motor impairment, reduction of intracellular α-Syn aggregates, suppression of microglial activation, reduced degeneration in the striatum, SNpc, Purkinje cells, pontine nuclei and inferior olivary complex
Fluoxetine (selective serotonin reuptake inhibitor)	MBP-α-Syn mouse model of MSA (Ubhi et al., 2012)	Amelioration of motor behavior, reduction of α-Syn aggregation, astrogliosis and demyelination, increased GDNF and BDNF levels, neuroprotection in the frontal cortex, hippocampus and basal ganglia
Fluoxetine	MBP-α-Syn mouse model of MSA (Valera et al., 2013)	Reduction of α-Syn aggregation in the basal ganglia, reduced astrogliosis in basal ganglia and hippocampus, modulation of proinflammatory and anti-inflammatory cytokines
Olanzapine		
Amitriptyline		
Mesenchymal stem cells	PLP-α-Syn mouse model of MSA (Stemberger et al., 2011a)	Relative preservation of SN TH-positive neurons, downregulation of the T-cell specific cytokines IL-2 and IL-17
	MPTP-3-NP double-toxin mouse model (Park et al., 2011)	Increased survival of dopaminergic neurons in the SN and striatum, anti-inflammatory and anti-gliotic effects

**Table 3:** Clinical trials of MSA.

	Intervention	Status	ClinicalTrials.gov identifier	Study design	No. of participants	Main outcome measure	Results
Completed	r-hGH (recombinant human growth hormone) (Holmberg et al., 2007)	Compl.		Phase II randomized, placebo-controlled	22 treated, 21 placebo	Safety, UPDRS, UMSARS, CAFTs	Trend to less worsening in UPDRS, UMSARS and CAFTs suggesting possibility of disease modification; higher dose and/or larger study group (>90) trials recommended
	Riluzole (NNIPPS) (Bensimon et al., 2009)	Compl.	NCT00211224	Phase II/III; randomized, placebo-controlled	194 treated, 197 placebo	Survival, mortality, different scales (*e.g.* UPDRS, NNIPPS-PPS)	No evidence of beneficial effect on survival and scales
	Minocycline (MEMSA) (Dodel et al., 2010)	Compl.	NCT00146809	Phase II randomized, placebo-controlled	31 treated, 31 placebo	UMSARS, UPDRS	No significant difference between treatment groups
	Rasagiline (Poewe et al., 2012)	Compl.	NCT00977665	Phase II; randomized, placebo-controlled	84 treated, 90 placebo	UMSARS DWI	No significant difference between treatment groups on the primary and secondary endpoints
	Lithium carbonate (Sacca et al., 2013)	Compl.	NCT00997672	Phase II randomized, placebo-controlled	4 treated, 5 placebo	Safety, UMSARS, micro- and macrostructural MR parameters	Terminated because of side effects
	IVIG (intravenous immunoglobulin) (Novak et al., 2012)	Compl.	NCT00750867	Phase II Open label	7 treated	Safety, efficacy	Safe, improved UMSARS scores
	Autologous mesenchymal stem cells (Lee et al., 2008b)	Compl.	Not listed	Phase I/II Open label	11 treated, 18 historical control group	Safety, UMSARS, FDG-PET	Safe, improved UMSARS scores compared to controls, increased FDG-uptake in cerebellum and white matter of the frontal cortex in the in the MSC-treated group compared to baseline Increased gray matter density in cerebellum
	Autologous mesenchymal stem cells (Lee et al., 2012)	Compl.	NCT00911365	Phase II; randomized placebo-controlled	14 treated, 17 placebo	UMSARS	Delayed progression in UMSARS I and II scores, less extensive decrease in glucose metabolism and gray matter density

Pending	Fluoxetine	Compl.	NCT01146548	Phase II randomized, placebo-controlled		UMSARS	Pending
	Rifampicin	Ongoing study	NCT01287221	Phase II/III; randomized, placebo-controlled		UMSARS	Pending

RCT, randomized controlled trial; FDG-PET 0, fluorodeoxyglucose PET; MSC, mesenchymal stem cells; PPS, Parkinson-Plus-Scale; CAFTs, cardiovascular autonomic function tests.

A double-blind, placebo controlled trial testing the effects of recombinant human growth hormone (r-hGH) has been performed by the European MSA Study Group (EMSA-SG), but no significant differences were found between placebo and r-hGH treated groups (Holmberg et al., 2007).

Studies in both rat unilateral and mouse systemic toxin models have indicated partial neuroprotection by the glutamate blocker riluzole (Diguet et al., 2005, Scherfler et al., 2005). There were no preclinical data on riluzole-mediated clearance effects of synuclein inclusions. The largest randomized controlled trial in MSA so far involving 398 subjects (NNIPPS (Neuroprotection and Natural History in Parkinson Plus Syndromes) study) revealed no significant differences in mortality and disease progression between placebo and active treatment groups (Bensimon et al., 2009).

Consistent with previous reports in the PD MPTP mouse model, Stefanova and co-workers detected evidence of neuroprotection in minocycline treated PLP-α-Syn MSA mice after early long-term treatment (Stefanova et al., 2007, Wu et al., 2002). Even though minocycline-treated patients also had reduced microglial activation on PK11195 PET analysis, the clinical trials for the treatment of MSA (NCT00146809) as well as PD were negative, most probably due to advanced disease stages at the time of study entry (Dodel et al., 2010, NINDS NET-PD Investigators, 2008).

Rasagiline, an irreversible MAO-B inhibitor, had neuroprotective effects against 3-NP in the PLP-α-Syn mouse model when applied before the neurotoxin (Stefanova et al., 2008). On this basis a phase II clinical trial of rasagiline as a potential MSA treatment was launched (NCT00977665), which did not show a significant effect of rasagiline over placebo on the primary and secondary endpoints (Poewe et al., 2012). Further analysis is ongoing. One critical issue in this study might also be the dose effect. The positive effects on motor behavior and neuronal survival in the transgenic mouse model were seen after treatment with high-dose (2.5 mg/kg) of rasagiline for four weeks. No significant effects were observed in mice receiving 0.8 mg/kg. In the trial MSA patients were assigned to low dose rasagiline due to safety concerns.

A randomized, double-blind, placebo-controlled study was performed to assess the safety and tolerability of lithium, a candidate neuroprotective therapy for MSA, however, the trial had to be terminated due to severe adverse effects (Sacca et al., 2013).

Intravenous immunoglobulin (IVIG) has been tested in a single-arm interventional, single-center, open-label pilot study and has been reported to be safe, feasible and well tolerated (Novak et al., 2012). A future randomized placebo-controlled trial will be required to evaluate the potential of IVIG as MSA treatment.

A phase II clinical trial has been launched evaluating the effects of fluoxetine in MSA patients, but results have not yet become available. Fluoxetine and other antidepressants have led to promising preclinical results including neuroprotection, amelioration of motor deficits and increased BDNF and GDNF levels, reduced accumulation of α-Syn and decreased astrogliosis in the MBP-α-Syn mouse model (Ubhi et al., 2012, Valera et al., 2013).

Rifampicin has been shown to prevent α-Syn aggregates from forming and to disaggregate already formed fibrils *in vitro* and in the MBP-α-Syn mouse model (Ubhi et al., 2008). A phase II clinical trial was launched to assess potential disease modifying effects of rifampicin in MSA, however, the investigators report that rifampicin did not slow or halt progression of MSA in rifampicin-treated patients (Low et al., 2014; Wenning et al., 2014).

Considering the lack of *SNCA* gene expression in oligodendroglia in controls and MSA, the uptake of extracellular α-Syn by oligodendroglia may indeed be pathologically increased in MSA (Miller et al., 2005). If CSF were to be the source of α-Syn, it would strongly suggest that antibody-based neutralization and capturing of α-Syn – ahead of its uptake by oligodendrocytes – could provide a plausible route to treatment. Studies using active immunization with hα-Syn and passive immunization with antibodies against different α-Syn epitopes have been performed in transgenic mouse models for PD and their positive results might also be relevant for evaluation in MSA models (Masliah et al., 2005, Masliah et al., 2011).

### 6.2 Cell based interventions

In contrast to neuroprotection, neurorestoration aims to replace neurons lost as a result of pathological events with new, functionally integrated neurons (Kollensperger et al., 2009, Lindvall and Kokaia, 2009, Lindvall and Kokaia, 2010, Puschban et al., 2000b, Wenning et al., 1996a). In MSA, two main types of cell-based therapies have been considered: (1) cell-based therapy for reversing l-dopa failure in MSA-P by embryonic striatal grafts into adult host striatum and (2) systemic mesenchymal stem cell delivery to slow disease progression.

#### 6.2.1 Cell based therapies for l-dopa failure

Animal studies have been performed to investigate whether the transplantation of embryonic striatal cells can restore dopamine receptor loss and lead to reversal of l-dopa failure, a key feature underlying progressive motor impairment in MSA-P. One of the first rat neurotoxin models of MSA was created in 1996 by the stereotaxic injection of 6-OHDA into the medial forebrain bundle, followed three to four weeks later by an injection of QA into the ipsilateral striatum (Wenning et al., 1996a). The investigators reported ipsilateral amphetamine and contralateral apomorphine rotations, with the latter being abolished after the QA lesion. However, the apomorphine-induced contralateral rotation behavior could be reversed by implantation of fetal CNS allografts consisting of cell suspensions from striatal primordium. This study was and still is of relevance to the field since it suggests that the unilateral MSA double lesion model could be turned into a PD-like model with graft-derived dopaminergic responsivity (Kollensperger et al., 2009, Puschban et al., 2000b×Wenning et al., 1996a). Comparing grafts in the PLP-α-Syn model with transplanted wt animals, reduced dopaminergic reinnervation and a pro-inflammatory environment due to α-Syn pathology or altered glial response have been reported in the transgenic animals, thus highlighting possible difficulties for future MSA transplantation trials (Stefanova et al., 2009b). Targeted striatal cell therapy for MSA may also be compromised by the progressive neurodegenerative process affecting brainstem, cerebellar and spinal cord regions.

#### 6.2.2 Systemic mesenchymal stem cell delivery

Another neurorestorative approach is the systemic delivery of autologous mesenchymal stem cells (MSCs) originally described by Lee et al., 2008b. In their open-label pilot study the authors compared MSA patients treated with MSCs, to untreated controls and found improvement in the treated group according to the unified MSA rating scale (UMSARS). However, the study design was suboptimal to address the safety and efficacy of MSC therapy in MSA (Quinn et al., 2008). Two experimental studies have generated preclinical evidence in favor of MSC therapy in MSA (Park et al., 2011, Stemberger et al., 2011a). Stemberger et al., 2011a reported a slight but significant recovery of SN tyrosine hydroxylase-immunoreactive neurons and a downregulation of the T-cell specific cytokines IL-2 and IL-17 after infusion of murine MSCs into the PLP-α-Syn model, probably due to peripheral effects, since most of the cells were trapped in the periphery and only a minute proportion found in the brain. Due to the disruption of the blood–brain-barrier in the MPTP + 3-NP mouse model, more MSCs were found in the brains in this study compared to the transplantation trial in the PLP-α-Syn model (Park et al., 2011). Park et al., 2011 observed increased survival of dopaminergic neurons in the SN, reduced microglial and astroglial activation and altered cytokine values after administration of human MSCs in an MPTP + 3-NP double-toxin MSA mouse model for MSA-P. Even though these promising results were obtained in a model of Parkinson variant MSA, based on these findings, a double-blind, randomized, placebo-controlled study with intra-arterial and repeated intra-venous injections of MSCs was performed in patients with the cerebellar variant of MSA, confirming the previous open-label results (Lee et al., 2012). Patients in the MSC group showed delayed progression in the UMSARS rating scale, as well as less extensive decrease in cerebral glucose metabolism and less severe decrease in gray matter density measured with FDG PET and MRI. Despite these positive effects reported by Lee and colleagues, MSC treatment is still considered an experimental approach and can thus not be recommended as treatment for MSA beyond the scope of properly conducted clinical trials. The significant difference between MSC intervention and treatment with placebo was based on a very low UMSARS score interpatient variability, much lower than those reported in previous natural history studies (May et al., 2007, Wenning et al., 2013) and previous MSA treatment trials, *e.g.* the r-hGH trial (Holmberg et al., 2007). This means that this study may have been negative in a control cohort reflecting published UMSARS score variations (Low and Gilman, 2012). Concerns have been raised about the safest and best standardized administration of MSCs since ischemic lesions have been reported in 29% of the MSC-treated patients. However, ischemic events were even more frequent (35%) in the placebo-treated patients, suggesting an angiography – rather than MSC-related complication (Lee et al., 2012, Low and Gilman, 2012). It has also been shown that intravenously applied MSCs are mainly trapped in the periphery and therefore it is still unclear whether at all the MSCs reach the brain and cross the blood–brain-barrier. Intrathecal application may be an alternative to intracarotid injection for enhancing delivery of MSCs to the brain, however, results of a retrospective case series in MSA and other parkinsonian disorders were negative (Storch et al., 2012). MSA-C patients have been exclusively enrolled into the Korean study, which is considered an additional weakness (Low and Gilman, 2012).

Many questions like the origin of administered cells, the cell type (stem cells, induced pluripotent stem cells or induced neurons directly converted from fibroblasts) or their optimal number per volume infused remain to be elucidated; these issues apply to the entire field of experimental CNS transplantation (Park et al., 2011, Pfisterer et al., 2011, Stemberger et al., 2011a, Takahashi et al., 2007).

## 7 Current bottlenecks for MSA therapy and how to overcome them

MSA research has progressed fast during the last decade due to multiple parallel developments; nevertheless, it is readily apparent that several obstacles need to be overcome to achieve cause-directed intervention, which would afford MSA patients with *bona fide* disease modification. Below, we list three of them.

One of the major limitations is the fact that MSA represents an orphan disease. Therefore, the number of study participants is limited and natural history studies or trials consistently run the risk of being underpowered. Several international MSA networks like the European, North American or Japanese MSA Study-Groups (EMSA, NAMSA, JAMSAC, NNIPPS) have been developed as a first step to overcome this problem. Due to MSA's orphan disease status it has rather low economic relevance for society and it is therefore difficult to obtain sufficient funds for preclinical drug development and clinically driven trials.

Two, disease recognition has improved over the last decade owing to validated consensus criteria (Gilman et al., 2008) and the development of diagnostic tools, in particular advanced MR imaging techniques (Mahlknecht et al., 2010, Mahlknecht et al., 2012, Meijer et al., 2013) that now serve as surrogate markers in clinical trials. However, the diagnostic inaccuracy in the early stages of MSA (Osaki et al., 2009) reminds us that improving the sensitivity of criteria by identification of motor and non-motor warning signs (red flags) and molecular neuroimaging methods is essential for meaningful progress to occur. Sofar, most interventional trials have been conducted in patients with probable MSA reflecting advanced disease stages with extensive neurodegeneration (Fig. 3). In the future, early stage patients with possible MSA in whom the diagnosis is supported by neuroimaging or other biomarkers should move into the focus of trial intervention.

Three, based on evidence of α-Syn mediated neurodegeneration in MSA, active and passive α-Syn immunization and α-Syn lowering compounds seem to be promising avenues. However, investigation of the *in vivo* α-Syn load requires development of accurate α-Syn imaging (Eberling et al., 2013). In addition, multiple studies have been performed aiming to develop suitable assays and identify CSF or plasma α-Syn assays for disease recognition and monitoring in α-synucleinopathies (Hall et al., 2012, Shi et al., 2010), however, the reported findings are both controversial and preliminary (Bidinosti et al., 2012, Constantinescu and Mondello, 2012, Mollenhauer et al., 2010, Mollenhauer et al., 2011, Mollenhauer et al., 2012).

## 8 Conclusion

Research on MSA has reached the starting point for a new era in attempted disease modification, *i.e.*, the planning of multicenter intervention trials; these should be based on evidence of target engagement in MSA models. Many preclinical and clinical studies have been performed in the past decade, searching for treatment agents that engage selective targets, as delineated in available disease models. To date, none of the clinical trials have produced the results we had hoped for based on the evidence gathered from pre-clinical studies. This is consistent with the failure of interventional therapies for other neurodegenerative disorders such as Parkinson's disease, Huntington's disease or motor neuron disease. In the future, patients need to be recruited in early disease stages and this will likely imply the use of imaging and wet biomarkers. Combination of PET and MR imaging might increase the diagnostic accuracy, however, to date there are few published data to support this. Further, evidence of target engagement for a given investigational compound will require functional imaging tools such as α-Syn PET ligands. To patients, their family members, basic science researchers and clinical practitioners, the quest for developing suitable treatment options for MSA patients continues to be fueled by the paucity of effective symptomatic therapy and the overwhelming absence of any disease-modifying intervention for this invariably fatal disorder. At the present, pre-clinical studies have identified α-Syn metabolism-directed compounds, including active or passive immunization against it, and systemic stem cell infusion as candidate therapies for the next generation of clinical trials. Undoubtedly, the execution of such trials will inform the development of therapies in the closely related disorder of typical PD, and *vice versa* since MSA and PD both have aggregates of misfolded α-Syn as major hallmark and share similarities in their pattern of neurodegeneration (Fellner et al., 2011, Fellner and Stefanova, 2012, McCann et al., 2014).

## 9 Search strategy and selection criteria

References for this Review were identified through searches on PubMed with the search terms ‘multiple system atrophy’ in combination with ‘etiology’, ‘pathology’, ‘pathogenesis’, ‘animal model’, ‘treatment’ and ‘transplantation’ from 1984 to December 2013 and on clinicaltrial.gov with the search terms ‘multiple system atrophy’. Scientific articles published in English language were reviewed exclusively. The final reference list was generated on the basis of originality and relevance to the topic.

## Authors’ contributions

DK-W undertook the literature search, analysis and screening for papers, wrote the manuscript, and prepared the figures. NS contributed to the writing, helped with the literature search and primary systematization, provided valuable input for the design of the figures and tables and helped with revisions. KAJ contributed largely to the pathology section. KS provided valuable input for the tables and helped with revisions. MGS was involved in the dissection of the literature and had large contributions to revisions. WP contributed to dissection of the literature and helped with revisions. GKW defined the search criteria, contributed to dissection of the literature, provided valuable input for primary systematization and figure design and the tables, and made the final revisions. All authors contributed significantly to the writing and corrections of this work and have seen and approved the final version.

## Conflicts of interest

There are no conflicts of interest.
